# Supplementary material for: Characteristics, outcomes, and mortality amongst 133,589 patients with prevalent autoimmune diseases diagnosed with, and 48,418 hospitalised for COVID-19: a multinational distributed network cohort analysis
Source: medRxiv. 2020 Nov 27:2020.11.24.20236802. Preprint. [Version 1] doi: 10.1101/2020.11.24.20236802 (PMC7709171; doi:10.1101/2020.11.24.20236802)
Supplement: Supplement 1 [file media-1.pdf]

Supplementary Figure 1. Database selection process

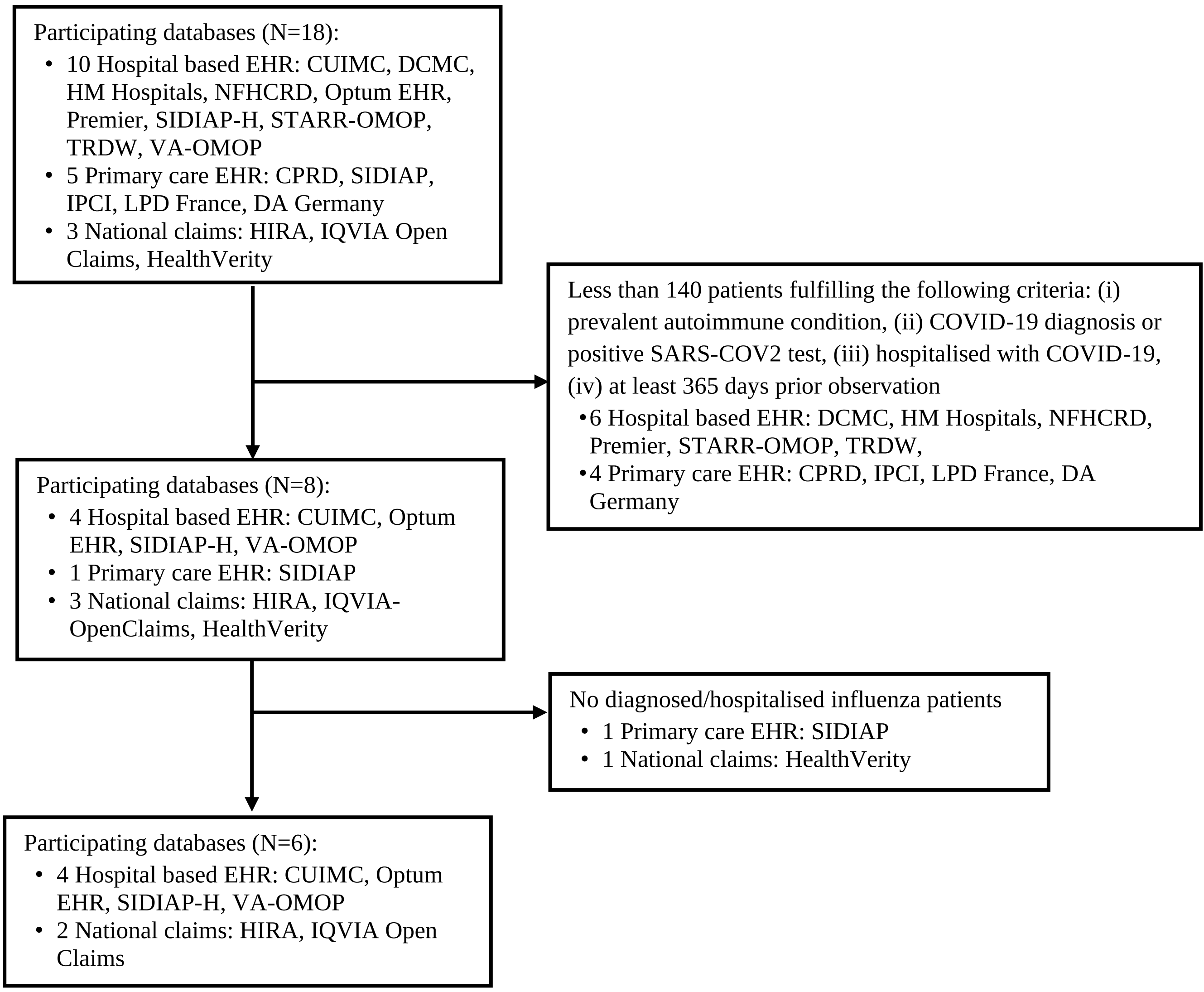

CPRD: Clinical Practice Research Datalink; CUIMC: Columbia University Irving Medical Center; DA Germany: IQVIA disease analyser Germany; DCMC: Daegu Catholic University Medical Center; EHR: Electronic health record; HIRA: Health Insurance Review & Assessment Service; LPD France: IQVIA Longitudinal Patient Data France; NFHCRD: Nanfang Hospital COVID-19 Research Database; IPCI: Integrated Primary Care Information; SIDIAP: Information System for Research in Primary Care; SIDIAP-H: SIDIAP– Hospitalisation Linked Data; TRDW: Tufts Research Data Warehouse, VA-OMOP: Department of Veterans Affairs

Supplementary table 1. Prevalence of autoimmune diseases in the year prior to the index date in patients diagnosed with COVID-19

The condition with the highest prevalence in most databases (highlighted in yellow) was reported as the prevalence for the respective autoimmune disease in Table 1 of the manuscript.

Autoimmune disease	Condition	CUIMC	HIRA	IQVIA-Open Claims	Optum EHR	SIDIAP-H	VA-OMOP
**Type 1 Diabetes Mellitus**	acidosis due to type 1 diabetes mellitus	0.0	0.0	0.0	0.3	0.0	0.0
	disorder due to type 1 diabetes mellitus	3.3	2.0	3.9	4.8	1.2	4.4
	gangrene due to type 1 diabetes mellitus	0.0	0.0	0.1	0.1	0.0	0.0
	hyperglycemia due to type 1 diabetes mellitus	1.9	0.0	1.9	2.9	<0.1	1.6
	hypoglycemia due to type 1 diabetes mellitus	0.5	0.0	0.4	0.8	0.0	0.4
	mild nonproliferative retinopathy due to type 1 diabetes mellitus	<0.4	0.0	0.1	0.3	0.0	0.3
	moderate nonproliferative retinopathy due to type 1 diabetes mellitus	0.0	0.0	0.1	0.1	0.0	0.2
	neuropathic arthropathy due to type 1 diabetes mellitus	0.0	0.0	0.0	0.1	0.0	0.0
	neuropathy due to type 1 diabetes mellitus	0.5	0.0	0.7	0.8	<0.1	0.6
	nonproliferative diabetic retinopathy due to type 1 diabetes mellitus	<0.4	0.0	0.2	0.4	0.0	0.5
	peripheral circulatory disorder due to type 1 diabetes mellitus	0.6	<0.6	0.4	0.4	0.6	0.2
	peripheral neuropathy due to type 1 diabetes mellitus	0.4	0.0	0.5	0.7	<0.1	0.4
	polyneuropathy due to type 1 diabetes mellitus	0.4	0.0	0.5	0.6	<0.1	0.4
	pre-existing type 1 diabetes mellitus	<0.4	0.0	0.0	0.1	<0.1	0.0
	pre-existing type 1 diabetes mellitus in pregnancy	<0.4	0.0	0.0	0.1	<0.1	0.0
	pregnancy and type 1 diabetes mellitus	<0.4	0.0	0.0	0.1	<0.1	0.0
	renal disorder due to type 1 diabetes mellitus	1.1	<0.6	0.9	1.2	0.3	0.6
	type 1 diabetes mellitus	3.4	1.5	5.8	6.0	5.0	4.4

	type 1 diabetes mellitus uncontrolled	0.0	0.0	0.0	0.8	0.0	0.0
	type 1 diabetes mellitus with arthropathy	0.0	0.0	0.0	0.1	0.0	0.0
	type 1 diabetes mellitus without complication	2.6	1.0	3.7	3.8	4.9	2.2
	ulcer of lower limb due to type 1 diabetes mellitus	<0.4	0.0	0.2	0.3	<0.1	0.3
**Rheumatoid arthritis**	myopathy due to rheumatoid arthritis	0.0	0.0	0.1	0.0	0.0	0.0
	polyneuropathy in rheumatoid arthritis	0.0	0.0	0.1	0.0	0.0	0.0
	rheumatoid arthritis	4.0	18.9	4.8	8.7	4.1	4.7
	rheumatoid arthritis - ankle and/or foot	<0.4	0.0	0.1	0.1	0.0	0.1
	rheumatoid arthritis - hand joint	0.5	0.0	0.2	0.5	<0.1	0.2
	rheumatoid arthritis of elbow	0.0	0.0	0.0	0.1	0.0	0.0
	rheumatoid arthritis of knee	<0.4	0.0	0.1	0.2	0.0	0.1
	rheumatoid arthritis of shoulder	0.0	0.0	0.1	0.2	0.0	0.0
	rheumatoid arthritis of wrist	0.0	0.0	0.1	0.1	0.0	0.1
	rheumatoid lung disease with rheumatoid arthritis	0.0	0.0	0.1	0.0	0.0	0.1
	seronegative rheumatoid arthritis	1.2	2.3	0.9	1.7	0.9	0.6
	seropositive rheumatoid arthritis	1.9	5.0	2.0	3.9	2.9	1.9
**Psoriasis**	arthritis mutilans	0.0	0.7	0.1	0.1	0.0	0.6
	generalized pustular psoriasis	0.0	<0.6	0.0	0.0	<0.1	0.1
	guttate psoriasis	<0.4	<0.6	0.0	0.2	1.9	0.2
	localized pustular psoriasis	0.0	<0.6	0.0	0.1	<0.1	0.0
	psoriasis	3.7	8.2	3.5	7.4	27.9	7.1
	psoriasis vulgaris	<0.4	2.8	1.3	1.0	0.3	2.6
	psoriasis with arthropathy	0.8	0.7	0.8	2.4	2.2	1.5
	psoriatic arthritis	0.0	0.0	0.0	0.1	<0.1	0.1
	psoriatic arthritis mutilans	0.0	0.0	0.1	0.1	0.0	0.6
	pustular psoriasis	0.0	0.9	0.1	0.2	<0.1	0.1
	pustular psoriasis of palm of hand	0.0	<0.6	0.0	0.1	<0.1	0.0
	pustular psoriasis of palms and soles	0.0	<0.6	0.0	0.1	<0.1	0.0
	pustular psoriasis of sole of foot	0.0	<0.6	0.0	0.1	<0.1	0.0

**Psoriatic Arthritis**	arthritis mutilans	0.0	0.7	0.1	0.1	0.0	0.6
	psoriasis with arthropathy	0.8	0.7	0.8	2.4	2.2	1.5
	psoriatic arthritis mutilans	0.0	0.0	0.1	0.1	0.0	0.6
**Multiple sclerosis**	multiple sclerosis	2.1	<0.6	2.2	3.3	2.2	1.9
**Systemic lupus erythematosus**	drug-induced systemic lupus erythematosus	<0.4	0.0	0.0	0.1	0.0	0.0
	lung disease with systemic lupus erythematosus	<0.4	0.0	0.0	0.1	0.0	0.0
	nephropathy co-occurrent and due to systemic lupus erythematosus	0.8	0.0	0.2	0.6	<0.1	0.1
	nephrosis co-occurrent and due to systemic lupus erythematosus	0.8	0.0	0.2	0.6	<0.1	0.1
	sle glomerulonephritis syndrome	0.8	0.0	0.2	0.6	<0.1	0.1
	systemic lupus erythematosus	3.4	1.7	1.9	3.6	2.3	1.1
	systemic lupus erythematosus with organ/system involvement	1.2	1.2	0.4	1.1	<0.1	0.2
	systemic lupus erythematosus with pericarditis	<0.4	0.0	0.0	0.1	0.0	0.0
**Graves' disease**	graves' disease	0.0	0.0	0.0	0.1	0.0	0.0
**Hashimoto thyroiditis**	hashimoto thyroiditis	0.0	0.0	0.0	2.1	0.0	0.0
**Myasthenia gravis**	myasthenia gravis	0.5	<0.6	0.4	0.6	1.0	0.6
	myasthenia gravis with exacerbation	<0.4	0.0	0.1	0.1	<0.1	0.2
	myasthenia gravis without exacerbation	0.4	0.0	0.4	0.6	1.0	0.5
**Vasculitis**	acute febrile mucocutaneous lymph node syndrome	<0.4	<0.6	0.0	0.1	<0.1	0.0
	acute hemorrhagic gastritis	0.0	6.9	0.3	0.3	<0.1	0.3
	antineutrophil cytoplasmic antibody positive vasculitis	<0.4	<0.6	0.1	0.2	<0.1	0.1
	arteritis	1.3	<0.6	0.7	1.1	3.5	0.6
	autoimmune vasculitis	<0.4	<0.6	0.0	0.2	<0.1	0.0
	behcet's syndrome	<0.4	0.9	0.0	0.1	0.4	0.1
	capillaritis	<0.4	<0.6	0.1	0.0	<0.1	0.1
	deep thrombophlebitis	<0.4	1.6	0.3	0.1	6.6	0.2
	deep vein phlebitis and thrombophlebitis of the leg	<0.4	1.6	0.3	0.1	6.6	0.2

	giant cell arteritis with polymyalgia rheumatica	0.0	0.0	0.0	0.0	<0.1	0.1
	granulomatosis with polyangiitis	<0.4	0.0	0.1	0.2	<0.1	0.1
	hypersensitivity angiitis	<0.4	<0.6	0.1	0.3	<0.1	0.0
	idiopathic capillaritis	<0.4	<0.6	0.1	0.0	<0.1	0.1
	necrotizing vasculitis	<0.4	<0.6	0.1	0.1	0.8	0.1
	phlebitis	2.1	4.7	2.6	3.6	12.8	1.8
	phlebitis and thrombophlebitis	0.7	1.6	0.9	1.1	10.2	0.6
	phlebitis and thrombophlebitis of intracranial sinuses	0.0	0.0	0.0	0.1	0.0	0.0
	phlebitis of deep veins of lower extremity	<0.4	1.6	0.4	0.2	6.6	0.3
	phlebitis of lower limb vein	1.5	2.9	2.0	2.6	9.2	1.3
	phlebitis of superficial veins of lower extremity	<0.4	<0.6	0.4	0.5	0.1	0.3
	phlebitis of the femoral vein	0.0	0.0	0.1	0.0	<0.1	0.1
	pigmented purpuric lichenoid dermatitis of gougerot and blum	<0.4	<0.6	0.1	0.0	<0.1	0.1
	polyarteritis	0.0	<0.6	0.1	0.1	<0.1	0.1
	polyarteritis nodosa	0.0	<0.6	0.1	0.1	<0.1	0.1
	primary systemic arteritis	<0.4	<0.6	0.1	0.1	0.2	0.1
	primary systemic vasculitis	0.5	1.7	0.2	0.5	0.6	0.3
	retinal vasculitis	<0.4	0.0	0.0	0.0	0.0	0.1
	rheumatoid vasculitis	<0.4	<0.6	0.0	0.1	<0.1	0.0
	secondary systemic vasculitis	<0.4	<0.6	0.1	0.2	<0.1	0.1
	small vessel vasculitis	<0.4	<0.6	0.2	0.4	0.2	0.2
	small vessel vasculitis caused by immune complex	<0.4	<0.6	0.0	0.2	<0.1	0.0
	systemic vasculitis	0.9	2.6	0.4	0.7	0.8	0.4
	temporal arteritis	<0.4	<0.6	0.3	0.4	0.8	0.3
	thromboangiitis	<0.4	<0.6	0.0	0.0	<0.1	0.1
	thromboangiitis obliterans	<0.4	<0.6	0.0	0.0	<0.1	0.1
	thrombophlebitis	0.7	3.3	0.9	1.2	10.2	0.6
	thrombophlebitis of deep veins of lower extremity	<0.4	1.6	0.3	0.1	6.6	0.2

	thrombophlebitis of lower extremities	<0.4	2.3	0.3	0.1	6.6	0.2
	varicose veins of lower extremity with inflammation	1.2	1.2	1.4	1.9	2.5	0.8
	varicose veins of lower extremity with ulcer and inflammation	<0.4	0.0	0.2	0.2	<0.1	0.2
	vasculitis	4.2	14.4	4.0	5.7	17.5	3.3
	vasculitis of medium sized vessel	<0.4	<0.6	0.1	0.1	0.2	0.1
	vasculitis of the skin	0.0	<0.6	0.1	0.2	0.1	0.2
**Pernicious anaemia**	pernicious anaemia	0.0	0.0	0.0	0.5	0.0	0.0
**Coeliac disease**	coeliac disease	0.9	<0.6	0.5	1.6	5.1	0.7
**Scleroderma**	crest syndrome	<0.4	0.0	0.1	0.1	0.0	0.0
	limited systemic sclerosis	<0.4	0.0	0.1	0.1	0.0	0.0
	localized scleroderma	<0.4	<0.6	0.1	0.5	0.3	0.1
	systemic sclerosis	0.6	<0.6	0.2	0.4	0.8	0.2
	systemic sclerosis with limited cutaneous involvement	<0.4	0.0	0.1	0.1	0.0	0.0
**Sarcoidosis**	cardiac sarcoidosis	<0.4	0.0	0.0	0.1	0.0	0.0
	cutaneous sarcoidosis	<0.4	0.0	0.0	0.1	<0.1	0.2
	lymph node sarcoidosis	0.0	0.0	0.0	0.0	<0.1	0.1
	pulmonary sarcoidosis	1.0	0.0	0.5	0.7	0.3	1.8
	sarcoid heart muscle disease	<0.4	0.0	0.0	0.1	0.0	0.0
	sarcoidosis	2.7	0.0	1.0	2.1	1.1	2.6
	sarcoidosis of lung with sarcoidosis of lymph nodes	<0.4	0.0	0.1	0.1	<0.1	0.3
**Ulcerative colitis**	abscess of intestine co-occurrent and due to ulcerative colitis	0.0	0.0	0.0	0.0	0.0	0.1
	chronic ulcerative colitis	0.6	0.0	0.4	1.2	0.1	0.2
	chronic ulcerative enterocolitis	0.0	0.0	0.0	0.1	0.0	0.0
	chronic ulcerative pancolitis	0.6	0.0	0.3	0.9	<0.1	0.2
	chronic ulcerative rectosigmoiditis	0.0	0.0	0.1	0.3	<0.1	0.1
	complication due to chronic ulcerative pancolitis	<0.4	0.0	0.1	0.2	0.0	0.1

	complication due to chronic ulcerative rectosigmoiditis	0.0	0.0	0.0	0.1	0.0	0.0
	complication due to ulcerative colitis	0.8	0.0	0.3	0.9	<0.1	0.8
	fistula of intestine due to ulcerative colitis	0.0	0.0	0.0	0.0	0.0	0.1
	intestinal obstruction due to ulcerative colitis	0.0	0.0	0.0	0.0	0.0	0.1
	left sided ulcerative colitis	<0.4	0.0	0.1	0.2	<0.1	0.4
	rectal hemorrhage due to chronic ulcerative pancolitis	<0.4	0.0	0.1	0.2	<0.1	0.0
	rectal hemorrhage due to chronic ulcerative rectosigmoiditis	<0.4	0.0	0.0	0.1	0.0	0.0
	rectal hemorrhage due to ulcerative colitis	<0.4	0.0	0.2	0.5	<0.1	0.7
	ulcerative colitis	1.9	<0.6	1.3	2.5	4.1	2.6
	ulcerative enterocolitis	0.0	0.0	0.0	0.1	0.0	0.0
	ulcerative pancolitis	0.6	0.0	0.3	0.9	<0.1	0.2
	ulcerative proctocolitis	0.0	0.0	0.1	0.3	<0.1	0.1
**Crohn's disease**	abscess of intestine co-occurrent and due to crohn's disease	<0.4	0.0	0.0	0.1	0.0	0.1
	abscess of intestine co-occurrent and due to crohn's disease of large intestine	<0.4	0.0	0.0	0.1	0.0	0.1
	abscess of intestine co-occurrent and due to crohn's disease of small and large intestine	<0.4	0.0	0.0	0.0	0.0	0.1
	abscess of intestine co-occurrent and due to crohn's disease of small intestine	<0.4	0.0	0.0	0.1	0.0	0.1
	complication due to crohn's disease	1.7	0.0	0.6	1.8	0.1	1.1
	complication due to crohn's disease of large intestine	1.2	0.0	0.3	1.0	0.0	0.6
	complication due to crohn's disease of small and large intestines	0.9	0.0	0.1	0.5	0.0	0.3
	complication due to crohn's disease of small intestine	1.3	0.0	0.3	0.9	<0.1	0.5
	crohn's disease	2.3	<0.6	1.2	3.0	2.9	1.6
	crohn's disease of intestine	1.4	<0.6	0.7	1.9	0.3	0.9
	crohn's disease of large bowel	1.0	0.0	0.4	1.3	<0.1	0.6

	crohn's disease of small and large intestines	0.5	0.0	0.2	0.5	0.0	0.4
	crohn's disease of small intestine	1.0	<0.6	0.5	1.2	0.2	0.7
	fistula of intestine due to crohn's disease of small and large intestine	<0.4	0.0	0.0	0.1	0.0	0.1
	fistula of large intestine due to crohn's disease	0.0	0.0	0.0	0.2	0.0	0.1
	fistula of small intestine due to crohn's disease	<0.4	0.0	0.0	0.1	<0.1	0.0
	gastrointestinal crohn's disease	1.4	<0.6	0.7	1.9	0.3	0.9
	intestinal obstruction due to crohn's disease	<0.4	0.0	0.1	0.3	<0.1	0.2
	intestinal obstruction due to crohn's disease of large intestine	<0.4	0.0	0.1	0.2	0.0	0.1
	intestinal obstruction due to crohn's disease of small and large intestine	<0.4	0.0	0.0	0.2	0.0	0.1
	intestinal obstruction due to crohn's disease of small intestine	<0.4	0.0	0.1	0.3	<0.1	0.2
	rectal hemorrhage due to crohn's disease	<0.4	0.0	0.1	0.2	0.0	0.1
	rectal hemorrhage due to crohn's disease of large intestine	<0.4	0.0	0.0	0.1	0.0	0.1
	rectal hemorrhage due to crohn's disease of small intestine	0.0	0.0	0.0	0.1	0.0	0.1

Supplementary table 2. Prevalence of autoimmune diseases in the year prior to the index date in patients hospitalised with COVID-19

The condition with the highest prevalence in most databases (highlighted in yellow) was reported as the prevalence for the respective autoimmune disease in Table 2 of the manuscript.

Autoimmune disease	Condition	CUIMC	HIRA	IQVIA-Open Claims	Optum EHR	SIDIAP-H	VA-OMOP
**Type 1 Diabetes Mellitus**	acidosis due to type 1 diabetes mellitus	<0.9	0.0	0.1	0.6	0.0	<0.2
	disorder due to type 1 diabetes mellitus	3.8	2.0	5.4	6.9	1.4	5.6
	gangrene due to type 1 diabetes mellitus	0.0	0.0	0.1	<0.2	0.0	<0.2
	hyperglycemia due to type 1 diabetes mellitus	2.7	0.0	2.4	3.7	<0.6	2.0
	hypoglycemia due to type 1 diabetes mellitus	<0.9	0.0	0.6	1.1	0.0	0.5
	mild nonproliferative retinopathy due to type 1 diabetes mellitus	0.0	0.0	0.2	0.2	0.0	0.3
	moderate nonproliferative retinopathy due to type 1 diabetes mellitus	0.0	0.0	0.1	<0.2	0.0	0.2
	neuropathic arthropathy due to type 1 diabetes mellitus	0.0	0.0	0.1	0.2	0.0	<0.2
	neuropathy due to type 1 diabetes mellitus	<0.9	0.0	1.0	1.5	<0.6	0.9
	nonproliferative diabetic retinopathy due to type 1 diabetes mellitus	0.0	0.0	0.3	0.4	0.0	0.6
	peripheral circulatory disorder due to type 1 diabetes mellitus	<0.9	<0.6	0.6	0.7	0.7	0.4
	peripheral neuropathy due to type 1 diabetes mellitus	<0.9	0.0	0.8	1.2	<0.6	0.8
	polyneuropathy due to type 1 diabetes mellitus	<0.9	0.0	0.7	1.0	<0.6	0.5
	pre-existing type 1 diabetes mellitus	0.0	0.0	0.1	0.3	0.0	0.0
	pre-existing type 1 diabetes mellitus in pregnancy	0.0	0.0	0.1	0.3	0.0	0.0
	pregnancy and type 1 diabetes mellitus	0.0	0.0	0.1	0.3	0.0	0.0
	renal disorder due to type 1 diabetes mellitus	1.4	<0.6	1.5	2.4	0.0	1.1
	type 1 diabetes mellitus	4.8	1.5	7.5	7.5	4.4	5.3

	type 1 diabetes mellitus uncontrolled	0.0	0.0	0.0	0.8	0.0	0.0
	type 1 diabetes mellitus with arthropathy	0.0	0.0	0.1	0.2	0.0	<0.2
	type 1 diabetes mellitus without complication	3.6	1.0	4.6	4.0	4.3	2.3
	ulcer of lower limb due to type 1 diabetes mellitus	<0.9	0.0	0.4	0.7	<0.6	0.7
**Rheumatoid arthritis**	myopathy due to rheumatoid arthritis	0.0	0.0	0.1	0.0	0.0	0.0
	polyneuropathy in rheumatoid arthritis	0.0	0.0	0.1	<0.2	0.0	0.0
	rheumatoid arthritis	4.8	18.9	4.9	8.8	5.4	4.0
	rheumatoid arthritis - ankle and/or foot	0.0	0.0	0.1	<0.2	0.0	<0.2
	rheumatoid arthritis - hand joint	<0.9	0.0	0.2	0.5	<0.6	<0.2
	rheumatoid arthritis of knee	<0.9	0.0	0.1	0.2	0.0	0.2
	rheumatoid arthritis of shoulder	0.0	0.0	0.1	0.3	0.0	<0.2
	rheumatoid arthritis of wrist	0.0	0.0	0.1	<0.2	0.0	<0.2
	rheumatoid lung disease with rheumatoid arthritis	0.0	0.0	0.1	<0.2	0.0	<0.2
	seronegative rheumatoid arthritis	1.3	2.3	0.9	1.3	1.4	0.5
	seropositive rheumatoid arthritis	1.8	5.0	1.9	3.6	4.5	1.9
**Psoriasis**	arthritis mutilans	0.0	0.7	0.1	<0.2	0.0	0.6
	guttate psoriasis	0.0	<0.6	0.0	<0.2	1.0	<0.2
	psoriasis	1.4	8.2	2.7	5.4	26.4	4.4
	psoriasis vulgaris	<0.9	2.8	1.0	0.5	<0.6	1.7
	psoriasis with arthropathy	0.0	0.7	0.6	1.7	2.5	0.9
	psoriatic arthritis mutilans	0.0	0.0	0.1	<0.2	0.0	0.6
	pustular psoriasis	0.0	0.9	0.1	<0.2	0.0	<0.2
**Psoriatic Arthritis**	arthritis mutilans	0.0	0.7	0.1	<0.2	0.0	0.6
	psoriasis with arthropathy	0.0	0.7	0.6	1.7	2.5	0.9
	psoriatic arthritis mutilans	0.0	0.0	0.1	<0.2	0.0	0.6
**Multiple sclerosis**	multiple sclerosis	1.1	<0.6	2.1	3.7	2.1	1.6
**Systemic lupus erythematosus**	lung disease with systemic lupus erythematosus	0.0	0.0	0.1	<0.2	0.0	<0.2
	nephropathy co-occurrent and due to systemic lupus erythematosus	1.1	0.0	0.3	1.0	<0.6	0.2

	nephrosis co-occurrent and due to systemic lupus erythematosus	1.1	0.0	0.3	1.0	<0.6	0.2
	sle glomerulonephritis syndrome	1.1	0.0	0.3	1.0	<0.6	0.2
	systemic lupus erythematosus	3.2	1.7	1.9	4.3	2.6	0.9
	systemic lupus erythematosus with organ/system involvement	1.4	1.2	0.5	1.5	<0.6	0.2
**Hashimoto thyroiditis**	hashimoto thyroiditis	0.0	0.0	0.0	0.9	0.0	0.0
**Myasthenia gravis**	myasthenia gravis	<0.9	<0.6	0.5	0.8	1.5	0.7
	myasthenia gravis with exacerbation	<0.9	0.0	0.2	0.2	0.0	0.3
	myasthenia gravis without exacerbation	<0.9	0.0	0.5	0.8	1.5	0.7
**Vasculitis**	acute hemorrhagic gastritis	0.0	6.9	0.4	0.4	<0.6	0.5
	antineutrophil cytoplasmic antibody positive vasculitis	<0.9	<0.6	0.2	0.4	<0.6	<0.2
	arteritis	<0.9	<0.6	0.8	1.8	3.8	0.9
	autoimmune vasculitis	0.0	<0.6	0.1	<0.2	<0.6	<0.2
	behcet's syndrome	0.0	0.9	0.0	<0.2	<0.6	<0.2
	capillaritis	0.0	<0.6	0.1	0.0	<0.6	<0.2
	deep thrombophlebitis	<0.9	1.6	0.3	<0.2	8.6	0.4
	deep vein phlebitis and thrombophlebitis of the leg	<0.9	1.6	0.3	<0.2	8.6	0.4
	granulomatosis with polyangiitis	<0.9	0.0	0.2	0.4	<0.6	<0.2
	granulomatosis with polyangiitis with multisystem involvement	<0.9	0.0	0.1	<0.2	0.0	<0.2
	hypersensitivity angiitis	0.0	<0.6	0.1	0.4	<0.6	<0.2
	idiopathic capillaritis	0.0	<0.6	0.1	0.0	<0.6	<0.2
	microscopic polyarteritis nodosa	0.0	<0.6	0.1	<0.2	0.0	<0.2
	necrotizing vasculitis	0.0	<0.6	0.1	<0.2	1.0	0.2
	phlebitis	1.8	4.7	2.8	4.7	15.5	2.3
	phlebitis and thrombophlebitis	<0.9	1.6	1.0	1.3	11.9	1.1
	phlebitis of deep veins of lower extremity	<0.9	1.6	0.5	0.3	8.6	0.5
	phlebitis of lower limb vein	1.1	3.0	2.2	3.5	12.2	1.6

	phlebitis of superficial veins of lower extremity	<0.9	<0.6	0.4	0.4	0.0	<0.2
	phlebitis of the femoral vein	0.0	0.0	0.2	<0.2	0.0	<0.2
	pigmented purpuric lichenoid dermatitis of gougerot and blum	0.0	<0.6	0.1	0.0	<0.6	<0.2
	polyarteritis	0.0	<0.6	0.1	0.2	0.0	<0.2
	polyarteritis nodosa	0.0	<0.6	0.1	0.2	0.0	<0.2
	primary necrotizing systemic vasculitis	0.0	<0.6	0.1	<0.2	0.0	<0.2
	primary systemic arteritis	0.0	<0.6	0.1	0.3	0.0	<0.2
	primary systemic vasculitis	<0.9	1.7	0.3	0.5	0.6	0.5
	rheumatoid vasculitis	<0.9	<0.6	0.1	<0.2	0.0	<0.2
	secondary systemic vasculitis	<0.9	<0.6	0.1	0.2	<0.6	<0.2
	small vessel vasculitis	<0.9	<0.6	0.3	0.4	<0.6	0.2
	small vessel vasculitis caused by immune complex	0.0	<0.6	0.1	<0.2	<0.6	<0.2
	systemic vasculitis	1.1	2.6	0.4	0.7	0.6	0.5
	temporal arteritis	<0.9	<0.6	0.3	0.7	1.0	0.4
	thromboangiitis	<0.9	<0.6	0.0	0.0	<0.6	0.2
	thromboangiitis obliterans	<0.9	<0.6	0.0	0.0	<0.6	0.2
	thrombophlebitis	<0.9	3.3	1.0	1.4	11.9	1.1
	thrombophlebitis of deep veins of lower extremity	<0.9	1.6	0.3	<0.2	8.6	0.4
	thrombophlebitis of lower extremities	<0.9	2.2	0.3	<0.2	8.6	0.4
	varicose veins of lower extremity with inflammation	<0.9	1.2	1.4	2.8	3.7	1.0
	varicose veins of lower extremity with ulcer and inflammation	<0.9	0.0	0.3	0.5	0.0	0.3
	vasculitis	3.4	14.4	4.4	7.7	20.8	4.4
	vasculitis of medium sized vessel	0.0	<0.6	0.1	0.2	0.0	<0.2
	vasculitis of the skin	0.0	<0.6	0.1	0.2	<0.6	0.3
**Pernicious anaemia**	pernicious anaemia	0.0	0.0	0.0	0.4	0.0	0.0
**Coeliac disease**	coeliac disease	<0.9	<0.6	0.3	0.9	1.2	0.4
**Scleroderma**	crest syndrome	<0.9	0.0	0.1	<0.2	0.0	0.0

	limited systemic sclerosis	<0.9	0.0	0.1	<0.2	0.0	0.0
	localized scleroderma	0.0	<0.6	0.0	0.2	0.6	<0.2
	systemic sclerosis	<0.9	<0.6	0.2	0.4	0.9	<0.2
	systemic sclerosis with limited cutaneous involvement	<0.9	0.0	0.1	<0.2	0.0	0.0
**Sarcoidosis**	cardiac sarcoidosis	<0.9	0.0	0.1	<0.2	0.0	<0.2
	cutaneous sarcoidosis	0.0	0.0	0.0	<0.2	<0.6	0.2
	pulmonary sarcoidosis	1.3	0.0	0.6	0.6	0.6	1.6
	sarcoid heart muscle disease	<0.9	0.0	0.1	<0.2	0.0	<0.2
	sarcoidosis	3.4	0.0	1.2	1.9	1.2	2.1
	sarcoidosis of lung with sarcoidosis of lymph nodes	<0.9	0.0	0.1	<0.2	<0.6	0.3
**Ulcerative colitis**	chronic ulcerative colitis	0.0	0.0	0.5	0.9	<0.6	<0.2
	chronic ulcerative pancolitis	0.0	0.0	0.4	0.7	<0.6	<0.2
	chronic ulcerative rectosigmoiditis	0.0	0.0	0.1	0.2	<0.6	0.0
	complication due to chronic ulcerative pancolitis	0.0	0.0	0.1	0.2	0.0	0.0
	complication due to ulcerative colitis	0.0	0.0	0.3	0.7	0.0	0.6
	left sided ulcerative colitis	0.0	0.0	0.1	0.2	<0.6	0.4
	rectal hemorrhage due to ulcerative colitis	0.0	0.0	0.1	0.2	0.0	0.4
	ulcerative colitis	<0.9	<0.6	1.3	2.2	2.8	1.6
	ulcerative pancolitis	0.0	0.0	0.4	0.7	<0.6	<0.2
	ulcerative proctocolitis	0.0	0.0	0.1	0.2	<0.6	0.0
**Crohn's disease**	abscess of intestine co-occurrent and due to crohn's disease	<0.9	0.0	0.1	<0.2	0.0	<0.2
	complication due to crohn's disease	<0.9	0.0	0.5	1.5	<0.6	1.0
	complication due to crohn's disease of large intestine	<0.9	0.0	0.2	1.0	0.0	0.6
	complication due to crohn's disease of small and large intestines	<0.9	0.0	0.1	0.4	0.0	0.3
	complication due to crohn's disease of small intestine	<0.9	0.0	0.3	0.8	0.0	0.4

	crohn's disease	1.1	<0.6	1.0	2.4	2.4	1.2
	crohn's disease of intestine	<0.9	<0.6	0.5	1.5	<0.6	0.7
	crohn's disease of large bowel	0.0	0.0	0.3	1.2	<0.6	0.4
	crohn's disease of small and large intestines	0.0	0.0	0.1	0.5	0.0	0.2
	crohn's disease of small intestine	<0.9	<0.6	0.4	1.0	<0.6	0.5
	fistula of large intestine due to crohn's disease	0.0	0.0	0.0	0.3	0.0	<0.2
	gastrointestinal crohn's disease	<0.9	<0.6	0.5	1.5	<0.6	0.7
	intestinal obstruction due to crohn's disease	<0.9	0.0	0.1	0.3	<0.6	0.2
	intestinal obstruction due to crohn's disease of large intestine	<0.9	0.0	0.1	<0.2	0.0	<0.2
	intestinal obstruction due to crohn's disease of small intestine	<0.9	0.0	0.1	0.2	0.0	<0.2
	rectal hemorrhage due to crohn's disease	0.0	0.0	0.1	0.4	0.0	<0.2
	rectal hemorrhage due to crohn's disease of large intestine	0.0	0.0	0.0	0.2	0.0	<0.2

Supplementary table 3. Severe outcomes and mortality in 30 days post hospitalisation in patients with COVID-19 and prevalent autoimmune diseases

Outcome	Cohort	CUIMC	HIRA	IQVIA Open Claims	Optum EHR	SIDIAP-H	VA-OMOP
**Acute kidney injury**	COVID-19	9.9	2.8	16.7	22.2	NA	31.1
	Influenza	29.7	3.9	11.9	16.4	5.3	26.3
**Acute myocardial infarction events**	COVID-19	2.5	2.6	2.4	6.3	NA	6.0
	Influenza	6.3	NA	3.4	4.1	<1.5	7.8
**Acute respiratory distress syndrome**	COVID-19	14.7	2.1	31.5	39.6	NA	42.8
	Influenza	17.1	NA	16.9	28.7	13.9	28.2
**Cardiac arrhythmia during hospitalisation**	COVID-19	12.4	3.8	13.6	29.5	NA	35.1
	Influenza	24.6	<3.3	17.0	28.1	19.2	32.0
**Death**	COVID-19	24.6	6.3	NA	NA	18.0	16.3
	Influenza	3.4	NA	NA	NA	2.2	4.3
**Heart failure during hospitalisation**	COVID-19	8.4	3.9	8.0	15.7	NA	24.5
	Influenza	35.4	5.2	14.1	20.5	14.9	28.3
**Pneumonia during hospitalisation**	COVID-19	12.6	40.7	45.7	53.2	NA	33.0
	Influenza	28.0	25.5	30.1	36.3	22.6	19.5
**Sepsis during hospitalisation**	COVID-19	4.7	4.9	17.3	23.5	NA	21.6
	Influenza	18.9	<3.3	16.4	21.1	3.7	21.4
**Stroke (ischaemic or haemorrhagic) events**	COVID-19	3.2	1.4	2.1	2.6	NA	3.4
	Influenza	<2.9	<3.3	2.0	2.6	<1.5	2.4
**Venous thromboembolic events**	COVID-19	3.2	1.4	3.3	7.6	NA	7.7
	Influenza	7.4	<3.3	3.0	4.1	2.5	4.6
